# The Bacteriophage Lambda CII Phenotypes for Complementation, Cellular Toxicity and Replication Inhibition Are Suppressed in *cII-oop* Constructs Expressing the Small RNA OOP

**DOI:** 10.3390/v10030115

**Published:** 2018-03-07

**Authors:** Karthic Rajamanickam, Sidney Hayes

**Affiliations:** Department of Microbiology and Immunology, College of Medicine, University of Saskatchewan, Saskatoon, SK S7N 5E5, Canada; kar029@mail.usask.ca

**Keywords:** bacteriophage lambda, CII regulation in lysogeny decision, OOP RNA

## Abstract

The temperate bacteriophage lambda (λ) CII protein is a positive regulator of transcription from promoter *pE*, a component of the lysogenic response. The expression of *cII* was examined in vectors devoid of phage transcription-modulating elements. Their removal enabled evaluating if the expression of the small RNA OOP, on its own, could suppress CII activities, including complementing for a lysogenic response, cell toxicity and causing rapid cellular loss of ColE1 plasmids. The results confirm that OOP RNA expression from the genetic element *pO-oop-to* can prevent the ability of plasmid-encoded CII to complement for a lysogenic response, suggesting that it serves as a powerful regulatory pivot in λ development. Plasmids with a *pO* promoter sequence of 45 nucleotides (*pO*45), containing the −10 and −35 regions for *oop*, were non-functional; whereas, plasmids with *pO*94 prevented CII complementation, CII-dependent plasmid loss and suppressed CII toxicity, suggesting the *pO* promoter has an extended DNA sequence. All three CII activities were eliminated by the deletion of the COOH-terminal 20 amino acids of CII. Host mutations in the *hflA* locus, in *pcnB* and in *rpoB* influenced CII activities. These studies suggest that the COOH-terminal end of CII likely interacts with the β-subunit of RNA polymerase.

## 1. Introduction

**Figure 1:**
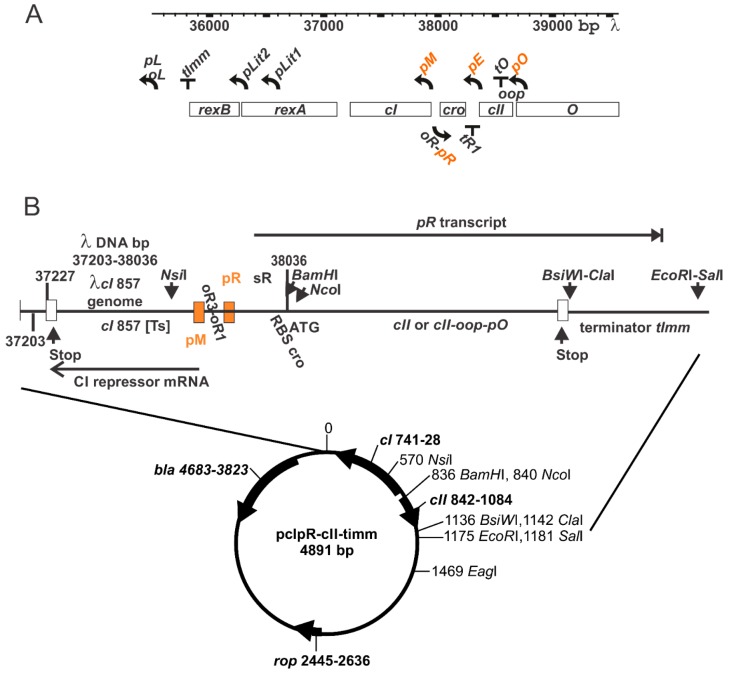
λ *imm*-region map and synthetic *cII* plasmid expression vector. (**A**) Map of the *imm*-*O* region of λ showing immunity region genes *rexB—cro*, several promoters (those relevant are shown in orange) and terminators, *tImm*, *tR1* and *tO*. The *cI* maintenance promoter, *pM* enables the expression of gene *cI*, encoding a repressor that binds to operators *oL* and *oR* and blocks transcription initiation from the major leftward and rightward promoters *pL* and *pR*. *pO* is required for transcription of the short OOP RNA [21]. The product of gene *cII*, transcribed from *pR*, stimulates repressor establishment transcription from promoter *pE*. The level of *pE* transcription can be 30- to 100-fold greater than the level of *cI* transcription from promoter *pM* upon thermal induction of a *cro* defective prophage [22,23,24] or 10- to 20-fold after λ infection [25]. *tImm*, terminates repressor establishment transcription from promoters *pM* and *pE*, preventing read-through into the *oL/pR* site. (**B**) Plasmid pcIpR-cII-timm (abbreviated herein as [cII] in tables) is a synthetic *cII* expression system (see Table S1 for related constructs) that eliminates gene *cro*, *ninR* and the *tR1* terminator, as shown in the plasmid insert (not drawn to scale). Gene expression from the *pR* promoter is regulated by the encoded temperature sensitive CI [Ts857] repressor, via binding to the *oR* operator sequences. Gene *cII* is positioned immediately downstream of the WT ribosomal binding site (RBS) for *cro*, which perfectly matches/can base pair with the 3′ terminal AUUCCUCCA sequence of 16S rRNA [26]. Potential stem-loop configurations in *pR* mRNA ahead of *cII* for the various plasmid constructs used are shown in Figure 2. Sequence variations (1–3) of pcIpR-cII-timm enabling *cII* expression arrangements are shown in Figure S1. The overall organization of genetic elements on plasmid pcIpR-[…]-timm was described [27,28,29], where a synthetic version of *tImm* is inserted between the *ClaI* and *EcoRI* sites to prevent read-through of transcription arising from promoter *pR*.

The regulatory circuits for control of the lysis-lysogeny decision made by bacteriophage λ upon infecting an *E. coli* host and the key involvement and modulation of CII activity have been extensively reviewed [1,2,3,4,5,6,7,8,9]. The elevated expression of gene *cII*, which is rightward and downstream from promoter *pR*, appears essential for lysogeny. CII serves as a positive transcriptional regulator to stimulate leftward transcription antisense to *cro* and sense for *cI* from promoter *pE* (Figure 1A), which is partially embedded within the first eight codons of *cII*. The enhanced expression of *cI*, encoding the CI repressor, results in its binding to operator *oL* (preventing leftward transcription from *pL*) and to operator *oR*, thus shutting down sR (rightward mRNA start site)-*cro-nutR-tR1-cII-O-P-ren…tR2-Q* transcription arising from *pR*. CII drives a lysogenic outcome by stimulating the transcription of *int* from promoter *pI* and antisense transcription of *Q* from promoter pAQ. The *cII* gene is regulated at multiple levels: by repressors CI and Cro binding at *oR* and limiting transcription from *pR* and by *nutR* and the *tRI* terminator site between *cro* and *cII* [1]. CII is degraded by the host ATP-dependent membrane protease FtsH (HflB), which is essential for cell growth [10,11,12]. This activity is modulated by the HflK-HflC proteins encoded by the *hflA* locus, which form a complex with FtsH [13], and by HflD [14]. The C-terminal 16 residues of CII are necessary and sufficient for rapid degradation by FtsH but are not sufficient for the activation of transcription by CII [15]. Proteolytic degradation of CII by HflB(FtsH)-HflC-HflK is inhibited by λ gpCIII [16,17,18]. At some level, the small RNA OOP serves to target the *cII* mRNA for degradation [19,20], although, the cleavage site for RNaseIII-dependent (and -independent) degradation is outside (12 nucleotides downstream) of the coding sequence for *cII* [20]. While suggesting a mechanism for OOP activity, these important studies measured CII activity via its ability to stimulate galactokinase expression from plasmid encoded *pE* and did not directly measure CII complementation reflected as limiting a lytic response from infecting phages. We show that OOP synthesis prevents CII-dependent stimulation of *pE* expression from infecting heteroimmune *imm*434 phages, limiting them to a lytic, rather than lysogenic, response.

Other aspects of CII function required further study. CII, as expressed from a multicopy plasmid, is highly toxic/lethal to *E. coli* [30] and this activity is ascribed to its inhibition of host cell DNA replication [31,32,33]. Localization of the toxic effect to sites within the CII protein have not been determined, nor has localization of the ability of CII to cause rapid loss of ColE1 plasmids, which is employed as an assay for replication inhibition. The influence of OOP RNA expression on CII toxicity has not been examined. The influence of OOP on CII complementation in trans, especially relative to OOP polyadenylation [34,35], required further examination. The influence of RNA polymerase mutations on CII activity and the impact of the high-frequency-lysogeny (Hfl) phenotype on CII complementation each required additional study. Finally, the genetics of *cII* expression is highly complex [36], with a large number of point mutations within and outside of the gene influencing its activity. Most of these mutations were rationalized based on the phenotype they conferred to an infecting λ phage and not independently evaluated synthetically. The effect of mRNA stem-loop formation immediately ahead of the ATG for *cII* (previously suggested to reduce ribosome attachment), as well as other mutations within and outside of *cII*, were examined and not surprisingly, mRNA structure ahead and within the N-terminal region of the *cII* transcript could influence *cII* expression. This work simultaneously assays for CII complementation, its cellular toxicity and the ability of CII to cause rapid loss of ColE1 plasmids. It examines the influence of *cII* linkage to *oop*, which amino acids of CII are required for complementation-toxicity-plasmid loss and the effect of host mutations in *rpoB*, the *hflA* locus and *pcnB* on CII activity. It explores the functional nucleotide length for *pO* activity and examines the effect of mRNA stem-loop formation ahead of the *cII* message and other mutations within and outside of *cII* on CII activities.

## 2. Materials and Methods

### 2.1. Strain Construction, Bacterial and Phage Strains Employed

Cellular growth medium, buffers, PCR amplification, DNA sequencing, transformation and P1 transduction methods were previously described [37], as were techniques for the construction of plasmid expression vectors for λ genes, gel analysis of plasmids, insertion localization and assays for plasmid loss and for cell viability resulting from the toxicity of plasmid-expressed λ genes [38]. *E. coli* strain employed: 594 [38], the *rpoB* mutants employed were isolated, mapped and sequenced in 594 [37]; *pcnB*::kan and *hflA*::kan alleles were moved by P1 from strains MG1655 *pcnB*::kan and X9368 *hflA*::kan (kindly provided by Drs. Grzegorz Węgrzyn and Susan Gottessman, respectively). Plasmids p27R and p27R*pO*^−^ (the basis for the synthetic *pO*38683-87MH mutation: 38683–38687-ATTAT to CGCGC) were previously described [39]. The *pO*38683LK mutation was synthetically generated using information from [40]. Phage λ*cI*72 DNA was used for amplifying λ gene segments for insertion into expression plasmids. Phages λ*imm*434*cII*68 (38498:T–A) and λ*imm*434*cII*2002 (38430:T–C) used for assaying CII complementation *in trans* were from the lab collection and are mutated in *cII* as reported [41]. The construction of pcIpR-D-GFPuv and visual assay for GFP expression was reported in [29].

### 2.2. Fluorescence Assays for Expressed D-GFPuv and GFPuv*

Cultures were inoculated from single cfu of 594 into LB and 594[pcIpR-D-GFP-timm] into Luria-Bertani (LB) broth (10 g Bacto tryptone, 5 g Bacto yeast extract, 5 g NaCl per liter) made 50 µg/mL with ampicillin = LB + Amp50 and grown overnight (ON) at 30 °C. Larger parallel subcultures (150 mL LB) were made by inoculating 7 mL from each ON culture to LB and then shaken at 30 °C until A_575_ = 0.2, whereupon they were moved into shaking water baths at 37, 39, or 42 °C. Aliquots were withdrawn prior to transfer and at 20 through 180 min post-induction (3 mL to measure absorbance and 5 mL was transferred and held on ice). Following the last sample removal, all the aliquots were centrifuged at 8000 rpm for five min, decanted and the cell pellets were re-suspended in 5 mL of 1× phosphate buffered saline (PBS) solution (10× = 80 g NaCl, 2 g KCl, 14.4 g Na_2_HPO_4_·2H_2_O, 2.4 g KH_2_PO_4_ per liter distilled H_2_O, pH 7.4), pelleted and re-suspended in 1 mL PBS. Three aliquots (100 µL) of each of the re-suspended cell pellets were assayed for fluorescence using a Photon Technology International, Model LPS 100, spectrophotometer (Horiba Instruments, Inc., Edison, NJ, USA). The excitation wavelength was set at 395 nm and emission fluorescence measured at 509 nm. Triplicate assays for each time point were made and the results averaged. Values for cells with plasmid represent the average of triplicate assays subtracting the triplicate average for cultures of 594 transferred in parallel to 37, 39 or 42 °C. (We appreciate that the fluorescence of cells grown in minimal medium is lower but the fluorescence assays for cells grown in LB were undertaken to parallel the other assay procedures employed herein.) To prepare cell lysates the cell pellets in 1 mL PBS were pelleted at 4500 rpm for 10 min and the supernatant was decanted. The pellet was re-suspended in 0.15 mL lysing mix (minus EDTA) made by adding 1.5 mL 1 M Tris·HCl, pH 7.8, 45 mL 1 M sucrose, 44.7 mL of distilled H_2_O and 0.8 mL of 10 mg/mL solution of lysozyme made in 10 mM Tris·HCl, pH 7.8. The cell suspension was incubated on ice for 30 min and the spheroplasts pelleted at 4500 rpm for 10 min and supernatant decanted. The pellet was suspended in 1 mL of 0.01 M Tris·HCl, pH 7.8 and allowed to sit 15 min at ambient temperature (~21 °C). The cell lysate was transferred into another tube and three 100 µL aliquots were measured for green fluorescence protein (GFP) activity as for the intact cell preps. The recorded fluorescence of the cell lysates was about half that observed from whole cells.

### 2.3. Complementation Assay for CII

Overnight LB + Amp50 cultures of cells with plasmids were mixed with LB top agar, overlaid on LB agar plates (in triplicate), then were spotted with 10^−6^, 10^−8^, or 10^−9^ dilutions of *cII* defective phages λ*imm*434 c*II*68 (lysate #433) and λ*imm*434 *cII*2002 (lysate # 717) made in buffer (0.01 M Tris HCl, pH 7.8, 0.01 M NaCl) and incubated for 24 h at 30°, 37° and 39 °C.

### 2.4. Determination of Toxicity and Plasmid Loss

This was done as previously described [37]. In brief, a single colony of *E. coli* cells with plasmid was inoculated in 20 mL of LB + Amp50 broth and incubated ON at 30 °C. The saturated cells were diluted in buffer, spread on LB plates and incubated at 30, 37, 39 and 42 °C. The colony forming units (cfu) on the incubated LB plates were counted and the cell viability was assessed by dividing the cfu titer on the 37, 39 or 42 °C-incubated plates by the titer of cfu arising on the plates incubated at 30 °C. Simultaneously, plasmid loss was evaluated by picking cfu from the 30, 37, 39 and 42 °C incubated plates and then stabbing them to fresh LB and LB + Amp50 plates that were incubated ON at 30 °C. The picked cfu that had lost the plasmid grew on the LB but not LB + Amp50 plates.

### 2.5. Oligonucleotides Used for Plasmid Construction and Sequencing

The oligonucleotides used for plasmid construction are shown in Table S2. General sequencing: L37904 + 18 (37,904–37,922 λ bp in *cI*) from *cI* and R-153-19 from backbone of pcIpR-[]-timm to the right of timm. Plasmid construction: L-Bam-CII (λ bp in *cII*: 38,359–38,394) and R-ClaBsi-CII (λ bp in *cII*: 38,619–38,650). These primers were used for inserting wild type *cII* but with an ochre, not wild type (WT) UGA stop codon. R-Bsi-45poWT encodes the wild type sequence between the start of *oop* at 38,675 through 38,769 in gene *O*. The expressed portion of *O* between 38,686–38,769 is truncated with a synthetic ochre stop codon after λ bp 38,769. This normal *O*-fragment amber stop codon was introduced in primers R-Bsi-45po38383LK and R-Bsi-45po38684-88MH. R-Bsi-45po38383LK includes the *pO*^−^ base change within “−10” region [40] at position 38,683, changing T to A. R-Bsi-45po38684-88MH changes the −10 sequence of *pO* as in [2] where bases 38,684–38,688 of the “−10” region sequence attatg/taatac (38,683–38,688) of *pO* were replaced by cgcgc/gcgcg. Primers for introducing COOH-terminal deletions within *cII*: R-Cla-Bsi-CII, R-Cla-Bsi-CIIΔ15, R-Cla-Bsi-CIIΔ30, R-Cla-Bsi-CIIΔ60, R-Cla-Bsi-CIIΔ90, R-Cla-Bsi-CIIΔ120 and R-Cla-Bsi-CIIΔ150. Other primers were used for introducing *cII* or *cII-oop-po* regions: L-BamXba38339 new was used to copy from λ WT sequence rightward from base 38,339–38,370 into *pE*(WT)-*cII*. R-noPo-Bsi-ClaI (38,618–38,653 bp λ), includes the same stop codon “TGA” as for WT *cII*, thus not altering the truncated *oop* sequence and was used to copy exactly *cII*, eliminating the portion of *oop* that does not overlap *cII* and the *pO* promoter region. R-Bsi-O-Po was used to add the 5′-end of *oop* sequence left of *cII* including the N-terminal end of *O* terminating with a synthetic ochre stop codon, including the *pO* sequence for *oop* with 39 bp homologous to the N-terminal end of gene *O*. Primers for introducing phage *cII* mutations 3638 and 3639 within the last six codons for *cII*, each with ochre *cII* termination codon introduced: R-Bsi-CII-3638 (methionine to valine, 4th amino acid encoded from end of *cII*), R-Bsi-CII-3639 (glutamine to arginine, 6th amino acid encoded from end of *cII*) and R-Bsi-3638 + 3639 (changes methionine to valine and glutamine to arginine in 4th and 6th amino acids from end of *cII*). Primers for introducing cy mutations (all silent): LXbaI-38339-cy3048, LXbaI-38339-cy2001, LXbaI-38339-cy42 and LXbaI-38339-cy3001. Primers LXbaI-38339-cy3048, LXbaI-38339-cy2001, LXbaI-38339-cy42 and LXbaI-38339-cy3001 were used with R-noPoBsiClaI (which eliminates *oop*) and R-Bsi-O-Po which includes *oop-pO*94. Primers used to make *oop-pO-O* containing plasmids for comparing their toxicity to *cII*-containing plasmids (see Table 7 in [5]): L-Bam-OOP#1, L-Bam-OOP#2, R-Cal-O, R-ClaI-36P and R-ClaI-63P.

### 2.6. Gene Expression Plasmid

Plasmid pcIpR, a precursor of pcIpR-[]-timm, was made by copying the *cI*857*-pM-oR1-3-pR* region of λ*cI*857 DNA with primers L-Mfe-37203 + 19 and R-Bam-38036-19 (numbers represent corresponding base pairs of λ genome) and inserting the 833 bp fragment between the *Eco*RI and *Bam*HI sites in pBR322. Inserting the *Mfe*I end into *EcoR*I nullifies both sites. As an intermediate, a synthetic 710 *Bam*H-*Sal*I fragment including codon optimized (Dcoe) D-(GGSGAP spacer-*Asc*I)-CAP-*Cla*I-timm-*Eco*RI-*Sal*I fragment [29] on plasmid pSMART (synthesized by Integrated DNA Technologies, Inc. [IDT], Coraville, IA, USA) was inserted into pcIpR, eliminating its *Bam*HI-*Sal*I segment to make pcIpR-D-CAP-timm [27,28], which includes the exact sequence of λ*cI*858 between bases 37,203 and 38,036 changing sequence and consensus RBS (underlined) left of the ATG for *cro* from λ38,029-TAAGGAGGT-TGT-ATG to 38,029-TAAGGAGGA-TCC-ATG, where ATG is the start of *cII*, not *cro*. In versions reported herein, *cII* expression units were cloned between *BamH*I and *Cla*I sites, eliminating the Dcoe-CAP-spacer segment.

## 3. Results

### 3.1. Thermally Inducible Gene Expression from pcIpR[]Timm Plasmid

**Figure 2:**
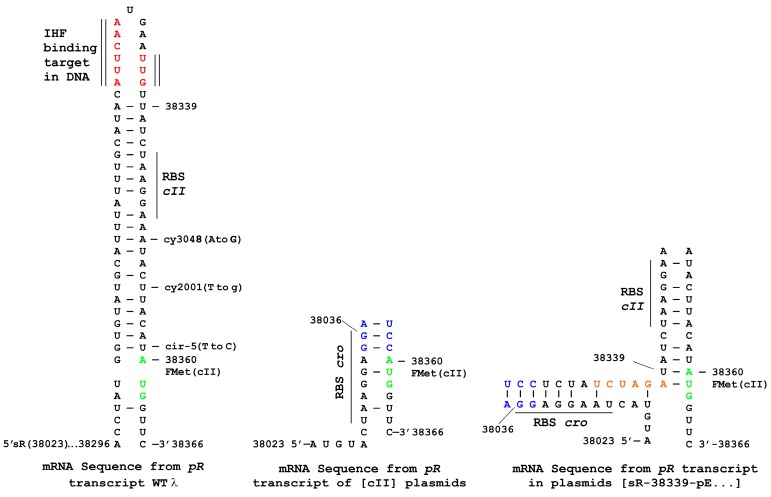
Potential stem-loop configurations in *pR* mRNA ahead of *cII* drawn from λWT and synthetic *cII* expression plasmids. In each structure, the start site for *pR*-initiated mRNA is suggested as arising at λ sequence base 38,302 just downstream from *pR* [48]. The structure on left omits the *cro-nutR-tR1* sequence and was inspired from a drawing in [36] showing the potential for stem loop mRNA secondary structure ahead of the translational start for *cII* that could impede translational initiation. The FMet codon for *cII* is green. An IHF (integration host factor) binding site in DNA [47] is shown in red which resembles the IHF consensus sequence AAAAAAnnnTTnnnWATCAAnnnTTR (where W is A or T, R is A or G and N is any nucleotide) but lacks the A-tract region about one helical turn from the consensus region [49]. The positions for some of the mutations [36] influencing *cII* that fall within the stem loop are shown. The structure in the middle includes the mRNA sequence produced from plasmid [CII]. This eliminates the stem-loop mRNA structure ahead of *cII* found in WT λ. It includes the WT λ sequence downstream from *pR* (bases 37,985–38,036 shown in Figure 1), the mRNA start site at 38,023 and the RBS for *cro* that is placed four bases ahead of the Fmet codon for *cII* (refer to Figure S1 and Materials and Methods). This arrangement included a base change equivalent to mutation cir-5 one base ahead of the FMet ATG starting at 38,360 of *cII*. The cir-5 mutation was suggested [36] to suppress the effect of mutation cII3086 at 38,360, which changes the ATG to GTG. The structure on the right includes plasmids that have the λ sequence for the 38,023 sR transcriptional start site downstream of *pR* and the WT λ DNA sequence downstream of base 38,339 (just downstream of the IHF binding site shown on figure at left), including the WT *pE* sequence. The bases for the *BamH*I (blue) and *Xba*I (pink) sites in middle and right drawings are shown as DNA sequences in Figure S1 (Plasmid groups 1 and 3, respectively).

In this report, plasmid-based synthetic *cII* constructs are transcribed from λ rightward promoter *pR*, which is regulated by a *cI* [Ts] repressor binding to *oR*, Figure 1 and which fully limits expression from *pR* at 30 °C. This plasmid does not include the *oL* operator so that octamer looping between the operators and CI repressor complex binding to produce a higher stability repression complex [42,43,44,45] is not possible. Transcription-influencing factors (*cro* expression, *nutR* and *tR1*) mapping between *pR* and *cII* were eliminated. This included removing an IHF (integration host factor) binding site (Figure 2) ahead of the RBS for *cII*. This site was suggested both to stimulate *cII* transcription by suppressing transcriptional termination at *tR1* [46] and to enhance *cII* mRNA translation by preventing the formation of a RNA:RNA duplex that includes the RBS in the message ahead of *cII* [47].

The expression of a gene cloned downstream from the promoter *pR* within plasmid pcIpR-[]-timm is influenced by culture temperature, Table 1. Previously, cultures of 594[pcIpR-GFP-timm] and 594[pcIpR-D-GFP-timm] (where GFP was fused downstream from λ gene D) were used to show that both cell types formed fluorescent cfu (colony forming units) when grown up on spread plates incubated at 37, 39 or 42 °C, compared to colonies grown up at 30 °C which did not fluoresce [29]. In these GFP plasmids the RBS for *cro* was positioned as described in Figure 1 for [cII], as drawn in the middle structure, Figure 2. Table 1 shows the kinetics of D-GFP fusion protein expression from *pR* upon shifting the culture temperature of 594[pcIpR-D-GFP-timm] cells growing at 30 °C to 37, 39 or 42 °C. GFP expression in cells raised to 37 °C was not observed until about 60–120 min post induction. The level of gene expression for cells raised to 42 °C by 150–180 min post induction was 43–75X that over cells raised to 37 °C and 8–10-fold over cells raised to 39 °C. By analogy, it is assumed that when *cII* is cloned downstream from promoter *pR*, its expression will resemble that of the D-GFP fusion protein. The ability of the Ts CI857 repressor encoded by the plasmid to reduce transcription from *pR* at 37 °C is lost at 42 °C. Limited *pR* gene expression at both 36 and 37 °C was previously observed in studies where the gene for the highly toxic P protein of λ was cloned downstream of *pR* in pcIpR-P-timm [38].

### 3.2. Untangling CII Activities: Cellular Toxicity and Promoting Plasmid Loss

The assay for CII toxicity measures if cells capable of expressing *cII* can survive and form cfu when grown in culture at 30 °C, then spread on plates incubated at 37, 39 or 42 °C. Per 1000 cfu of 594[cII] cells spread on plates incubated at 39° or 42 °C (Table 2), respectively, on average, only 30 or 1 cfu were observed. Cellular toxicity and plasmid loss is dependent on CII production. In CII_1–92_, deletion of the last five amino acids restores cell viability by 24-fold (0.73/0.03) at 39 °C. Deletion of the terminal 10 amino acids (in CII_1–87_) prevents plasmid loss at 42 °C. Deletion of the terminal 20 amino acids (in CII_1__–__77_) completely suppressed CII toxicity and its ability to stimulate plasmid loss. (Identical results were observed for CII_1__–__67_, CII_1__–__57_ and CII_1__–__47_ as for CII_1__–__77_.) Thus, the last 10 amino acids of CII are involved in plasmid loss. The last 5 amino acids significantly influence CII-dependent cellular toxicity.

Two cII missense mutations cII-3639 and cII-3638, that respectively change the 6th (cII mutation 38634 Gln to Arg) or 3rd (mutation 38642 Met to Val) amino acid from the COOH-terminal end [36], did not modulate CII toxicity but reduced plasmid loss at 42 °C. Combining the two mutations on one *cII* gene greatly reduced plasmid loss, i.e., 94% plasmid retention at 42 °C, suggesting an involvement of the six C-terminal amino acids of CII in plasmid loss.

### 3.3. CII Complementation

**Figure 3:**
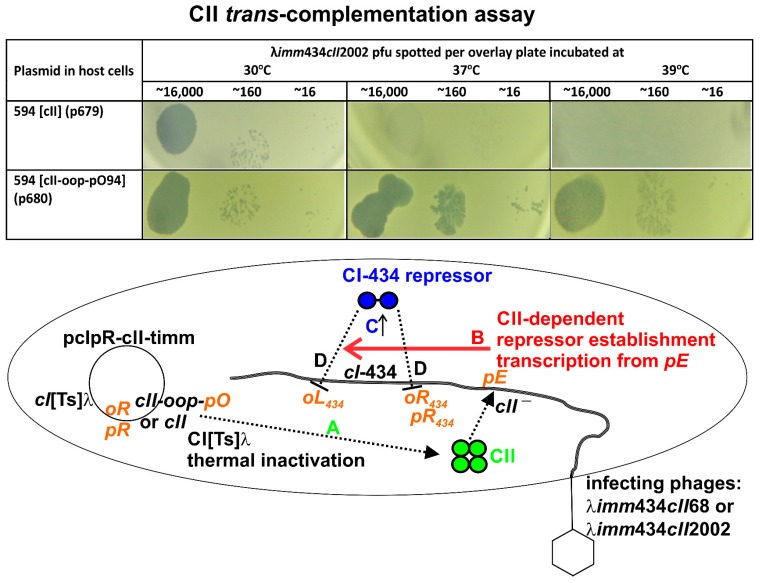
CII *trans*-complementation assay. Top shows complementation results (additional examples are shown in Figures S2 and S3). The cartoon shows the sequential steps, A to D required for CII complementation. Each step is indicated by either dotted (steps A and D) or solid (steps B and C) lines. Steps: A *cII* expression occurs from plasmid pcIpR-cII-timm in 594 cells upon shifting cells grown at 30 °C to 37–42 °C. This inactivates the CI_λ_ repressor, enabling transcription from plasmid promoter *pR* through gene *cII*. B CII protein encoded by plasmid stimulates repressor-establishment transcription from promoter *pE* on phage genome after 594 [pcIpE-cII-timm] cells are infected with phage λ*imm*434*cII*2002. C High expression of the CI_434_ repressor protein occurs from CII-stimulated *pE*-mRNA transcribed from infecting phage genome. D CI_434_ repressor binds the *oL*_434_ and *oR*_434_ operator sites preventing leftward and rightward transcription and blocking lytic phage growth. The potential for OOP RNA expression from plasmid pcIpR-cII-oop-pO nullifies CII-activation of *pE*-dependent CI_434_ expression at 37–39 °C. The results for infections with λ*imm*434*cII*68 were identical to infections with λ*imm*434*cII*2002.

The assay for CII complementation, Figure 3, measures the expression and ability of plasmid cloned *cII* to stimulate, in trans, repressor establishment transcription from promoter *pE* encoded on two infecting heteroimmune *imm*434 lambdoid phages mutated for *cII* (cII68, 38498: T-A, Trp to Arg; cII2002, 38430: T-C, Leu to Pro). Each hybrid phage includes the WT λ sequence rightward from λ base 38265 left of *cII*, including an identical IHF binding site, RBS for *cII*, the −10 and −35 sequences of *pE* and reported regions of extended dyad symmetry [50]. CII-stimulated, *pE*-dependent CI_434_ repressor establishment transcription from the infecting phage genome confers a high level of *cI*-434-repressor expression. The CI_434_ can bind to the *imm*434 *oL* and *oR* operators on the infecting phage. This blocks phage transcription from the *imm*434 *pR* promoter, lytic phage replication and growth and arrests plaque formation at 37 °C or 39 °C, effectively shunting the infecting *cII*-defective phage into a non-vegetative (pre-lysogenic) state. Even weak *cII* expression from [cII] at 37 °C was sufficient to prevent plaque formation by the infecting *imm*434 phages (Figure 3). Clear plaque forming units (pfu) arose in cell lawns incubated at 30 °C due to the repression of *cII* expression from [cII]. Plasmids deleted in *cII* for five or ten COOH-terminal amino acids (respectively cII_1–92_ and cII_1–87_), or those with *cII* mutations cII-3638, cII-3639 and cII-3638 + 3639 fully complemented for CII activation of repressor establishment transcription from *pE* (Table 2, Figure S2). Plasmid cII_1–77_ and those with larger terminal deletions failed to complement for CII activity. Thus, either the last 20 amino acids, or those 11 to 20 from the C-terminal end are required for CII activation of transcription from *pE*.

Contrary to an expectation that plaque formation would be inhibited, *hflA* cells with plasmids [cII], [cII_1–92_] and [cII_1–87_] allowed weak activation of transcription from *pE* but did not block phage growth, Table 2, Figure S2. Plasmid [cII_1–77_] was fully defective for CII complementation in *hflA*::kan cells as clear pfu were formed at 37–39 °C, Table 2. Significantly, the *pcnB*::*kan* mutation, shown to be a null allele for *pcnB* [51], completely abrogated CII complementation activity at 37 and 39 °C (Table 2, Figure S2). These results strongly implicate an involvement of poly (A) polymerase I in CII *complementation* that was not noted previously.

Cells with transduced rifampicin-resistant mutations in *rpoB*, encoding the β-subunit of *E. coli* RNA polymerase, were examined to determine if a CII-RpoB interaction was involved in CII complementation, cell toxicity, or plasmid loss. In the *rpoB* mutant BI (Table 3), CII expression from plasmids [cII] and [cII_1–92_] (but not [cII_1–87_]) yielded high level complementation, blocking lytic phage growth at 39 °C. The remaining *rpoB* mutants (B8, C1, C4, C10, D2, D6) interfered with CII complementation, Table 3. This result supports the conclusion that a CII-RpoB interaction participates in CII complementation. The loss of any CII complementation by [cII_1–87_] in five of seven *rpoB* mutants suggests that an interaction between the terminal ten amino acids of CII and RpoB is an essential component of CII-stimulated *pE* transcription.

### 3.4. Host Modulation of CII-Dependent Cellular Toxicity and Plasmid Retention

The host null mutation *hflA*::kan reduced CII toxicity at 39 °C (30-fold-compare viabilities of 0.03 and 0.89) and at 42 °C, Table 2. CII expression was highly toxic in 594 *pcnB*::*kan* cells, even at 39 °C (Table 2).

Several of the *rpoB* mutants, B1 (Q148P), B8 (L571Q), C4 (ΔGEV440-442V), D2 (R451S) and D6 (G537C) had been shown to confer cellular resistance to the highly toxic λ gpP, while C1 (P564L) and C10 (R529H) remained P-sensitive [37]. Except for B1, both P^R^ and P^S^ mutants, suppressed CII toxicity at 39 °C (cell viabilities: 594[cII], 0.03; *rpoB*[cII]: B1, 0.003; B8, 0.65; C1, 0.96; C4, 0.15; C10, 0.14; D2, 0.95; D6, 0.11) and 42 °C. Mutants C1 and D2 were highly protective. The P^R^ mutants C4, D2 and D6 reduced or eliminated CII-dependent plasmid loss. These results suggest that CII:RpoB participates in cellular CII toxicity and plasmid loss at 42 °C (Table 4). The mutant C4 exhibited high toxicity without plasmid loss, suggesting CII toxicity and plasmid loss arise via different actions of CII.

### 3.5. Influence of oop RNA Expression on cII-Dependent Cellular Toxicity and Plasmid Loss

The *oop* RNA gene is expressed leftward from promoter *pO* and terminates at the hairpin formed at the end of *oop*, designated the *tO* terminator (Figure 1). 52-bases of the oop sequence overlap the C-terminal region of cII (Figure S1). Plasmid loss: *oop* expression from [cII-oop-pO94] eliminated CII-dependent plasmid loss at 42 °C in WT 594 cells (Table 2) and in *rpoB* mutants B1, B8, C1 and C10 (Table 4). It was unable to do so in 594 cells with null mutations *hflA*, *pcnB* (Table 2) or the *rpoB* mutants D2 and D6 (Table 4)*.* CII-toxicity: *oop* expression suppressed CII toxicity at 39 and 42 °C in strain 594 (Table 2) and in the *rpoB* mutants B1, B8, C4 and by >50–250 fold in *rpoB* mutants C1 and C10, respectively (Table 4)*.*

### 3.6. Influence of Terminally Overlapping Divergent cII and oop Transcription on CII Complementation

Several [cII-oop-pO] plasmid constructs were examined herein. *oop* expression from [cII-oop-pO94], abrogated CII complementation in 594 WT and the 594 *hflA* cells at 39 °C (Table 2)*.* In [cII-oop-pO94] the promoter for *oop* is within the 94 bp to the right of the 5′end of the OOP RNA. The putative sequence for *pO* was predicted in the original sequence determined by Scherer et al. [52] to include a −10 region between 8 and 13 nucleotides rightward from base 38,675 encoding the 5′ end of the OOP RNA and a −35 region (Figure S1, plasmids 2). An attempt was made to establish a minimal sequence encoding *pO* and to assess the effect of introduced mutations within the −10 for *pO*. Plasmids were constructed that included the predicted WT −10 and −35 regions ahead of the actual start of *oop* transcription, i.e., variations of [cII-oop-pO45] and versions that included the −10 mutations LK (A to T at 38,683) [40] or MH (i.e., changing bases 38,683–38,687, ATTAT to CGCGC) [39]. The WT pO45 (p763) and both the pO45 −10 mutant plasmids (p759, p762) fully complemented for CII-activated CI_434_ expression at 37–39 °C (Table 5, Figure S3), suggesting there was no interference of CII complementation and little if any *oop* expression by the pO45 plasmids. The −10 position for *pO* suggested by Scherer et al. [52] appears correct since introduction of the MH mutation into the pO94 plasmid (p681) allowed for full CII complementation, i.e., for CII-activated CI_434_ expression at 37–39 °C. Thus, the −10 mutation MH nullified *oop* expression, at least in comparison to that from WT [cII-oop-pO94] (Table 2). These data suggest that unrecognized upstream nucleotides between 45 and 94 from the 5′ end of *oop* contribute to the function of *pO*.

### 3.7. Plasmid Construction Variations and Influence on cII Expression

The WT *pR* mRNA can form a stem-loop structure ahead of the amino-terminal region of *cII* (Figure 2) [36,53]. It was proposed [36] that this stem-loop structure reduces the rate of translation initiation, possibly by restricting the access of ribosomes to the initiation region and that the binding of IHF to this region was essential for the translation of *cII* mRNA [47]. Several plasmid variants were generated (Figure S1, plasmids 3), including ones with upstream WT bases 38,339 through the *cII* mRNA start codon. These encoded the potential for mRNA stem-loop formation occluding the ribosomal binding site (RBS bases 38,343-38,348) ahead of AUG for *cII* (Figure 2) but not including the IHF binding site, nor most of the region of dyad symmetry present in the WT *cII* mRNA [47]. These plasmids encoded the −10 and −35 regions of *pE* (respectively, bases 38,350–38,355 outside of *cII* and the dual CGTT sequences encoded by bases 38,369–38372 and 38,379–38,382 within the amino terminal end of *cII*). Some of them included the pO94 sequence, or four point mutations believed to inactivate *pE*, i.e., mutations cy3048 or cy2001 within −10 and cy42 or cy3001 within each of the CGTT sequences in −35.

Several differences were apparent when comparing data for plasmids with/without the 38,339–38,360 sequence that could form a stem-loop structure. Plasmid [cII] was 21-fold more toxic in cells raised to 39 °C than [sR-38339-pE-cII] (Table 2 and Table 5), suggesting the formation of a stem-loop can reduce *cII* expression. In contrast, the ability of *oop* expression from plasmid [cII-oop-pO94] to reduce CII toxicity at 42 °C (Table 2) decreased by 8-fold for [sR-38339-pE-cII-oop-pO94] (Table 5) and suppression of plasmid loss at 42 °C no longer occurred. This suggests stem-loop formation ahead of the *cII* message can influence activities involving pO94.

Adding −10 region mutations cy3048 or cy2001 (Figure S1**)** to [sR-38,339-pE-cII] reduced CII toxicity by 10 to 80-fold, and, even without *oop*, they completely suppressed CII-dependent plasmid loss at 42 °C (Table 5). The −35 region mutation cy3001 reduced plasmid loss and cy42 eliminated CII toxicity at 42 °C. The plasmid [sR-38,339-pE-cII] with added cy mutations 2001, 42, or 3048 poorly complemented in trans for CII (Table 5), permitting clear plaque formation by the infecting *imm*434*cII* phages at 37 °C and weak- turbid pfu at 39 °C. (The cy3048 mutation was included in plasmids described in [47]). Plasmid [sR-38339-pE-cII] with cy3001 complemented better, suppressing plaque formation at 39 °C, yet supporting individual turbid plaque formation at 37 °C.

It was assumed that neither the point mutations (cy3048, cy2001) introduced ahead of *cII* in the −10 for *pE* nor the silent mutations (cy42 and cy3001) within *cII* in the −35 of *pE* would influence CII complementation in trans. This assumption proved wrong, as CII complementation by [sR-38339-pE-cII] was altered by the putatively cis acting cy mutations.

## 4. Discussion

The studies reported here both confirm and extend several of the observations made by Kobiler et al. [15], who demonstrated CII_1–82_, deleted for the terminal 15 amino acids, was degraded very slowly by FtsH, compared to CII WT, suggesting the terminal amino acids of the CII monomer represent an important target for FtsH proteolysis and thus CII protein stability within the cell. But they concluded that the terminal 16 amino acids were not required for CII activity, i.e., CII_1–81_ was functional, CII_1–80_ was non-functional. These results parallel our finding a CII_1–77_ protein deleted for the COOH-terminal 20 amino acids was unable to complement for CII. However, we found that the terminal amino acids of WT CII_1–97_ can influence its ability to complement, its toxicity and its effect on plasmid stability in a WT host. Whereas, CII_1–92_ and CII_1–87_ were as active as CII_1–97_ for CII complementation, CII toxicity was reduced by 20-fold in CII_1–92_ and plasmid loss was fully suppressed in CII_1–87_. Thus, the C-terminal end of CII is not without activity in a WT host. Deletion of the C-terminal 10 residues in CII_1–87_ eliminated complementation in hosts with five distinct point mutations in *rpoB*, each conferring rifampicin resistance, suggesting that these residues influence CII complementation.

The CII complementation assay employed here assumed that *cII* expression from a plasmid would stimulate, in trans, *pE* transcription from infecting *imm*434 heteroimmune phages, each with a nullifying *cII* mutation. No complementation is observed at 30 °C, since there is no *cII* expression. But, expressing a high level of CI_434_ repressor will shut down *imm*434 *pL* and *pR* transcription from the infecting phage, blocking plaque formation (lytic phage growth), without influencing *cII* expression encoded on the *imm*λ plasmid. This assay measured two components of CII activity. Trace levels of *cII* expression were expected at 37 °C (and hence turbid plaque formation but not inhibition of lytic growth). Intermediate levels of *cII* expression were expected at 39 °C (compared to 42 °C with fully constitutive *cII* expression), turbid plaque formation and perhaps plaque inhibition. It was found: (i) constructs exhibiting weak CII complementation supported turbid or weak-turbid plaque formation at 37 and 39 °C; (ii) cells defective for CII complementation gave clear pfu at 37 and 39 °C; and (iii) cells with full CII complementation prevented plaque formation at both 37 and 39 °C. Kobilier et al. [15] suggested that CII inhibits lytic phage functions perhaps through an effect on phage DNA replication and/or Q gene expression. The results for high CII complementation, do not distinguish between (i) CII action to stimulate very high CI_434_ expression that can shut down phage transcription, from (ii) an activity of CII that independently and perhaps non-specifically, inhibits lytic growth, e.g., by inhibiting replication, or by any other mechanism. There are some hints to tease apart the two potential actions of CII, each manifested by the inhibition of phage plating and to make sense of mutations arising within and outside of *cII* that influence CII activity.

Extrinsic factors modulate the link between high CII complementation and high CII toxicity. Early studies identified host *hflA* and *hflB* mutations (conferring high frequency of lysogeny phenotype) that enable λ^+^ and λ*cIII* mutants to lysogenize these cells at high frequencies [54], suggesting higher CII activity in these cells. What seems undisputed is that the FtsH (HflB) ATP-dependent membrane protease degrades CII [15] and that the HflK-HflC proteins encoded by the *hflA* locus can individually inhibit the FtsH-mediated proteolysis of CII in vitro [16]. When it was examined whether an *hflA*::kan null mutant would support enhanced CII degradation, 30-fold *less* CII toxicity was observed at 39 °C. This suggested that inactivating the *hflA* locus reduces CII toxicity and its interference with lytic phage growth, which is more in keeping with its effect on FtsH and that CII toxicity can be a factor in λ development. We propose that by reducing some form of CII toxicity, e.g., via enhanced CII degradation, the frequency of λ lysogeny is increased. This is an alternative explanation for the HflA phenotype to one based on enhancing CII activity.

What is responsible for CII toxicity? It was shown that the single base substitution G537C responsible for *rpoB* D2 [37] can completely suppress CII toxicity and plasmid loss at the highest level of *cII* expression at 42 °C (with cell viability of 0.95 at 39 °C and no plasmid loss), while reducing but not eliminating CII complementation at 39 °C. This suggests that both CII toxicity and plasmid loss depend upon a binding interaction between CII and *E. coli* RNA polymerase holoenzyme, or a direct interaction with the β-subunit encoded by *rpoB*. The *rpoB* D2 mutation also confers resistance to cell killing by the highly toxic λ gpP. Even trace expression of *P* significantly exceeds the replication-inhibition and plasmid curing levels conferred by CII. For example, minimally expressed *P* from plasmid pcIpR-P-timm in cells grown at 36 °C [38] cured the cells of ColE1 plasmids. Hammer et al. previously isolated *E. coli rpoB* mutants S531F and P564L resistant to killing by CII protein [55]. One mutant used, *rpoB* C1 (P564L), shared an identical mutation as reported by Hanmer et al. [55] but was not nearly so resistant to CII as were the *rpoB* D2 cells.

Kedzierska et al. [31] found that *E. coli* growth inhibition was independent of mutations in CII (A30T) [56] or in *rpoA* (L271E) [57] that abolish transcriptional activation by CII [58] at several positively regulated promoters [59]. They suggested that CII toxicity is independent of its transcription activation function, even given the potential for 294 CII binding sites noted to be present in the *E. coli* genome [31]. They rationalized, because of the almost immediate effect of CII overproduction, that its inhibitory effect to both *E. coli* and λ replication was linked to interference with the DNA replication process. Herein, an assay for cellular loss of ColE1 plasmids was employed as an indicator for replication inhibition. The *rpoB* mutations D2 (R451S) and C4 (Δ:GEV440-442V) completely abolished plasmid loss when *cII* was constitutively expressed at 42 °C and mutation D6 (G537C) was almost as effective. These results suggest that the inhibitory effect of CII on plasmid maintenance involves its interaction and interference at a stage of RNA polymerase participation in replication.

RNA transcripts terminated by the *oop* terminator, *tO* are reported very labile, compared to transcripts from other terminators [60]. The influence of polyadenylation on *cII* and *cII-oop* expression was examined, since it was suggested that polyadenylation by *E. coli* poly (A) polymerase I (PAP I) leads to destabilization of RNA in bacterial cells [61,62]. Reports suggest that OOP RNA is polyadenylated by PAP I, that this is impaired in a *pcnB* mutant [35] and that polyadenylated OOP RNA is rapidly degraded [34] in comparison to non-polyadenylated OOP. CII activity in cells with a *pcnB* null mutation was examined. [Note that *cII*-mRNA expression terminates at *tImm* from [cII] missing *oop-pO*]. Surprisingly, all CII complementation activity was suppressed, the *imm*434*cII* phages plated efficiently (suggesting any CII made was not inhibiting phage growth) and yet high-level cellular CII toxicity and plasmid loss were evident. It would appear, that cellular capacity for polyadenylation is essential for some aspect of CII complementation. Coupling *cII-oop* expression could reverse cellular toxicity at 39 °C but not plasmid loss. This was a first indication that the state of *cII*-mRNA can influence CII complementation.

The results illustrate a profound nullifying effect of *oop* expression from promoter *pO* on CII complementation, toxicity and plasmid loss, suggesting emphatically that OOP is a regulatory pivot in λ development and that *oop* transcription can abrogate CII activity and drive the lytic response. This effect occurred in the *lexA*^+^ cells employed, where a LexA repressor binding sequence within the *oop-pO* sequence [63] can potentially limit *oop* expression. RNaseIII-dependent and independent cleavage of a cII-mRNA:OOP RNA hybrid was suggested as a mechanism by which OOP RNA could modulate CII-mRNA expression. Both cleavage sites are outside of *cII* [20] and thus secondary nucleolytic degradation of the *cII*-mRNA would be required to inactivate the message, for which the efficiency has not been reported.

The −10 and −35 regions of *pO* were suggested from the original DNA sequence of this region [52] and are fully contained within *pO*45 that includes 45 nucleotides upstream from the 5′-end of *oop* sequence. It can be argued from CII toxicity and plasmid loss data at 42 °C that there is some *oop* expression from *pO*45, since mutations at LK (38,683) and MH (38,683-87) in −10 suppressed this but the plasmid cII-oop-pO45WT still retained a high level of CII complementation. CII complementation was completely defective in cells with the cII-oop-pO94 plasmid but was restored in cells with plasmid cII-oop-pO94MH with an altered −10 region of *pO*. This data supports, both that the −10 region is correctly identified and that extending *pO* an additional 49 bases rightward into the *O* sequence from *pO*45 to *pO*94 profoundly influences *cII* expression/complementation, all other factors being equivalent. The *tO-oop-pO* transcriptional unit was identified from thermally induced defective prophage [21] deleted for all cell lysis and morphogenesis genes rightward from genes *O-P-ren*. The level of *oop* RNA synthesis increased 15^+^ fold following de-repression of replication competent prophage but remained static after de-repression of eight strains defective in λ replication initiation [64], including prophage with *ori*λ defects. These and other studies suggested there was no direct correspondence between the level of OOP RNA synthesis and the number of λ DNA copies per cell. Because the *oop* sequence was found to be dispensable for λ growth [65] (as is *cII*), questions relating to whether *oop* expression is regulated, or is merely gene dosage dependent have remained unsolved. It is unknown if the OOP RNA made from a multicopy plasmid equates to that made from an induced prophage. None of the plasmids employed herein were capable of *ori*λ-dependent replication initiation. Thus, the difference between *oop* expression from *pO*45 and *pO*94 most likely depends upon a transcriptional up-regulation signal for *oop* located in the additional *O* gene sequence present in *pO*94.

Based on these studies, it is suggested that assays measuring the global cellular effect of CII should include a functional *cII-oop-pO* construct. In this report, plasmid-expressed CII complemented, in trans, *pE* transcription initiation from infecting *cII*-defective *imm*434 phages. An opposite assay was employed to assess *cII* expression from infecting phages, where the CII made stimulated, in trans, *pE* transcription from a plasmid devoid of *oop* and apparently a promoter for *cII*. In these assays [8,66] the phage encoded CII activity stimulated *pE* dependent GFP or mCherry expression from ColE1 plasmid pSA11 with λ coordinates 38,336–38,653 [67]. The λ segment on the plasmid was deleted for the IHF binding site ahead of *cII* but included the stem-loop structure ahead of *cII* up through five amino acids from its COOH-terminal end, eliminating the 5′ end of *oop* and its *pO* promoter. It was assumed there was little/no *cII* expression from the plasmid borne λ segment, only from the infecting phages. The possible influence of *pO* transcription on *pE* expression from the plasmid remained unexamined.

In this work, plasmids were made that could support the potential for stem-loop formation, Figure 2 (structure on right) ahead of *cII* and they conferred reduced CII toxicity and likely reduced *cII* expression. The potential for stem-loop formation ahead of *cII* reversed some of the activities involving *oop* expression from promoter *pO*94. Accordingly, point mutations were introduced within the stem-loop but outside of *cII*. They also reduced CII toxicity and suppressed its effect on plasmid loss. Amazingly, the single silent mutation, cy42 in codon four of *cII*, within *pE*, completely suppressed CII toxicity at 42 °C. These observations require further study to gain understanding but strongly suggest that the hitherto un-explored state of *cII*-mRNA structure (excluding the IHF binding site and regions of dyad symmetry ahead of *cII*) remain an important component in *cII* expression.

Finally, it is unclear if CII can stimulate *pE* transcription from every DNA template, e.g., can it express *pE* from a template where *cII* is being transcribed? In discussing a voting rule for lysogeny, Zeng et al. [66] found there was no *pE* activity in cells defective for *dnaJ*, which is required for λ replication. Long ago, an enormous difference was reported [23] between CII- and CIII-dependent *pE* expression following the induction of a λ*cI*857*cro*27 prophage in a WT host that supported the replication of an induced λ prophage, compared to the induction of the same prophage in a *dnaB*[Ts] host that prevented λ replication and from which there was no detectable *pE* transcription. For the WT cells, there was a 10- to 15-fold increase in DNA copies of the λ*cI*857*cro*27 prophage by about 25 min post induction [22]. It remains unclear if the increased number of λ templates were responsible for the large difference in *pE* expression between replication competent and deficient conditions.

## 5. Conclusions

1. The results confirm that OOP RNA expression from the genetic element *pO-oop-to* can suppress high CII activity and that OOP RNA likely serves as a powerful regulatory pivot in temperate lambdoid phage development.
2. Plasmids with a *pO*94, comprising 94 bases rightward from *oop*, prevented CII complementation, CII-dependent plasmid loss and suppressed CII toxicity, suggesting that the active *pO* promoter to produce OOP requires an extended DNA sequence, beyond that required to encode the −10 and −35 regions.
3. All three CII activities were eliminated by the deletion of its COOH-terminal 20 amino acids.
4. *E. coli* mutations were shown to influence CII activities. (a) Inactivating the *hflA* locus encoding HflK-HflC proteins that modulate the FtsH ATP-dependent membrane protease significantly reduced CII trans-complementation and toxicity; (b) A null allele of *pcnB*, encoding poly (A) polymerase I, eliminated CII complementation and increased CII toxicity; (c) Five of six *rpoB* point mutations significantly reduced CII trans-complementation; (d) The CII_1–87_ mutant, deleted for the terminal 10 amino acids, lost its ability to complement in five *rpoB* mutant cells.
5. The results suggest that the terminal end of CII likely interacts with the β-subunit of RNA polymerase.

## Figures and Tables

**Table 1:** Temperature dependence of thermally inducible intra-cellular D-GFP fusion expression from plasmid pcIpR-D-GFP-timm [fluorescence units (±SE) × 10^−3^].

Induction Time (min)	Culture up-Shift from 30 °C ± SE ^a^		
	37 °C	39 °C	42 °C
0	0 (0.2)	0 (4.0)	3.2 (0.05)
20	0 (0.2)	1.4 (2.6)	153.8 (0.5)
40	0 (0.3)	6.7 (2.0)	415.4 (0.4)
60	0.8 (0.3)	23.2 (3.2)	518.5 (0.5)
90	0 (0.3)	41.3 (0.3)	624.6 (0.4)
120	7.3 (14.9)	30.0 (2.9)	721.2 (0.4)
150	10.8 (1.3)	84.3 (3.4)	808.4 (0.4)
180	23.9 (2.6)	136.9 (10.7)	1039.5 (0.7)

D-GFP is a fusion protein generated by fusing the DNA sequence for green fluorescence protein to DNA sequence for λ capsid decoration protein D. ^a^ All data represent triplicate determinations averaged. Then the triplicate average for up-shifted 594 cells (to 37, 39, or 42 °C for the indicated times) was subtracted from the triplicate averaged upshifted 594[pcIpR-D-GFP-timm] whole cells. The standard error (SE) values represent the average of SE values for determinations of 594 cells with or without a plasmid.

**Table 2:** Influence of C-terminal *cII* amino acids and host properties on *cII* complementation, toxicity and plasmid loss.

Host [Plasmid] ^a^	Intensity of CII-Activated CI_434_ Repression at 37–39 °C ^b^	λ*imm*434 *cII*^−^ Plaque Formation at 37–39 °C ^c^	Cell Viability, (±SE) and [% Plasmid Loss] Per Growth Temp. of Transformants ^e^	
			39 °C	42 °C
594 [cII] = [cII_1–97_]	H (high)	−	0.03 (0.001) [0]	<0.001 (0.0001) [100]
594 [cII-oop-pO94]	0 (none)	+ ^d^	0.59 (0.03) [0]	0.08 (0.03) [0]
594 [cII_1–92_]	H	−	0.73 (0.01) [0]	0.005 (0.001) [100]
594 [cII_1–87_]	H	−	0.76 (0.02) [0]	0.08 (0.03) [0]
594 [cII_1–77_]	0	+ ^d^	0.74 (0.10) [0]	0.32 (0.10) [0]
594 [cII-3638](38,642: A-G, M-V)	H	−	0.62 (0.10) [0]	0.002 (0.0004) [89]
594 [cII-3639](38,634: A-G, Q-R)	H	−	0.76 (0.02) [0]	<0.001 (<0.0001) [19]
594 [cII-3638–3639]	H	−	0.65 (0.04) [0]	<0.001 (<0.0001) [6]
594 *hflA*::kan [cII]	S (slight)	+	0.89 (0.01) [0]	0.008 (<0.0001) [100]
594 *hflA*::kan [cII_1–92_]	S	+	0.69 (0.01) [0]	<0.001 (<0.0001) [100]
594 *hflA*::kan [cII_1–87_]	S	+	0.85 (0.02) [0]	0.24 (<0.0001) [0]
594 *hflA*::kan [cII_1–77_]	0	+ ^d^	0.86 (0.10) [0]	0.58 (0.01) [0]
594 *hflA*::kan [cII-oop-pO94]	0	+ ^d^	0.63 (<0.0001) [0]	0.001 (0.0002) [100]
594 *pcnB*::kan [cII]	0	+ ^d^	<0.001 (0.01) [67]	<0.001 (0.0001) [100]
594 *pcnB*::kan [cII-oop-pO94]	0	+ ^d^	0.46 (0.01) [64]	<0.001 (0.0003) [89]

^a^ All strains were made by transformation of an Amp^R^ plasmid into strain 594, or into 594 cells that had been transduced with *hflA*::kan or *pcnB*::kan markers. All CII protein deletions were from the COOH-terminal end, where the WT CII is represented as cII_1–97_. ^b^ Intensity of CII-activated CI repression depends upon CII expression from the plasmid stimulating *pE*-*cI*^434^ transcription from an infecting λ*imm*434*cII*^−^ phage genome. “H” indicates a high level of CII complementation of the *cII*-defective infecting phage by the plasmid-encoded *cII* allele. The high level of CI^434^ expression from the phage genome represses lytic development, preventing the formation of individual plaques and phage lysis spots on overlay plates incubated at 37 and 39 °C. “0” indicates that no CII complementation was detected and the infecting phage plated, forming clear plaques, at an equivalent efficiency to that observed on overlaid 594 host cells without a plasmid. “S” indicates slight CII complementation, insufficient to block lytic phage growth and plaque formation and yielding weakly turbid plaques, suggesting the CII activity was not sufficient to drive establishment *pE-cI*^434^ transcription and CI^434^ repressor synthesis to a level that could prevent lytic growth of the infecting λ*imm*434*cII*^-^ phages. ^c^ Plaquing designations: “−” indicates that no individual plaques were observed in spots of 16 or 160 pfu and faint or no lysis was evident when 16,000 pfu were spotted. “+” indicates cell lysis when 16,000 pfu were spotted and individual pfu formation in each of the spot areas with applied 160 or 16 pfu. ^d^ Individual plaques were clear. ^e^ Measurements for plasmid loss: a minimum of 36 cfu per assay plate per plating temperature were screened, in triplicate. The assay was also conducted in parallel (for every experiment shown herein) at 30 °C, where there is no *cII* expression due to the fully active λ CI Ts857 repressor and at 37 °C; and for each assay, the viability was 1.0 with no plasmid loss.

**Table 3:** Influence of *rpoB* mutations on CII complementation.

Host(s) Strains ^a^	CII-Construct Plasmid(s) with Thermally Inducible Expression of *cII* Allele	Intensity of CII-Activated CI_434_ Repression at 37–39 °C ^b^	λ*imm*434 *cII*^−^ Plaque Formation at 37–39 °C ^b^
594 *rpoB* B1	cII; cII_1–92_	H	−
594 *rpoB* B8, C1, C4, C10, D2, D6	cII; cII_1–92_	S	+
594 *rpoB* B1, C1	cII_1–87_	S	+
594 *rpoB* B8, C4, C10, D2, D6	cII_1–87_	0	+ ^c^

^a^ *rpoB* mutations in 594 host, P^R^: B1 Q148P, B8 L571Q, C4 Δ:GEV440-442V, D2 R451S, D6 G537C (each confer resistance to the toxic λ gpP protein); P^S^: C1 P564L, C10 R529H. P^R^ and P^S^ indicate which *rpoB* mutations confer resistance, or sensitivity to λ P protein lethality. ^b^ Designations as in Table 2. ^c^ Individual plaques were clear. The designations H, S, 0, for intensity of CII activated repression were as noted in Table 2.

**Table 4:** Suppression of CII toxicity and plasmid loss by *oop* expression in *rpoB* mutants.

Host Strains with *rpoB* Alleles	Cell Viability (±SE) [% Plasmid Loss] Per Growth of Transformant at 42 °C	
	[cII] ^a^	[cII-oop-pO94] ^b^
594 ^c^	<0.001 (0.001) [100]	0.08 (0.03) [0]
594 *rpoB* B1	<0.001 (<0.0001) [100]	0.07 (0.01) [0]
594 *rpoB* B8	0.01 (<0.0001) [78]	0.13 (0.03) [0]
594 *rpoB* C1	0.007 (0.10) [100]	0.36 (<0.0001) [0]
594 *rpoB* C4	0.003 (0.010) [0]	0.07 (<0.0001) [0]
594 *rpoB* C10	0.004 (0.001) [100]	1.00 (0.01) [0]
594 *rpoB* D2	0.38 (<0.0001) [0]	0.10 (<0.0001) [81]
594 *rpoB* D6	0.02 (0.01) [6]	0.03 (0.01) [86]

^a^ Plasmid includes precise sequence (ATG to TTC) for *cII* cloned into pcIpR-cII-timm in the same position in plasmid, relative to *pR* and the *sR* start site for rightward transcription as is gene *cro* in λWT, including its RBS sequence, except for substitution of a BamHI site cloning site incorporated into the RBS, eliminating the natural DNA sequence leftward (upstream) of *cII*, which includes a stem-loop structure [36] that could influence *cII* expression. The *cII* sequence in [cII] terminates with an ochre rather than TGA stop codon, Figure S1. ^b^ Plasmid includes the same sequence as in [cII] but with natural amber termination signal for *cII*, plus the natural partially *cII*-overlapping *oop* sequence, plus 94 nucleotides rightward of the start point (38685) for 5′ end of *oop* transcription, that includes the *pO* promoter for *oop*, through base 38769 in gene *O*. This encodes 28 amino acids from the N-terminal end of *O*, terminating with an inserted ochre stop codon, Figure S1 plasmids 2. ^c^ Data from Table 2 repeated here for comparison.

**Table 5:** Expression variations for *cII* in 594 host.

CII-Construct Plasmids with Thermally Inducible Expression of *cII* Allele	Intensity of CII-Activated CI_434_ Expression at 37–39 °C ^a^	λ*imm*434 *cII*^−^ Plaque Formation at 37–39 °C ^a^	Cell Viability (±SE) [% Plasmid Loss] Per Growth Temp. of Transformants	
			39 °C	42 °C
sR-38339-pE-cII (p747)	H	−	0.62 (0.10) [0]	<0.001 (<0.004) [94]
sR-38339-pE-cII-oop-pO94 (p748)	0	+ ^d^	0.82 (0.02) [0]	0.001 (<0.0001) [94]
sR-38339-pE-cII-cy3048 in −10 *pE* (p767)	0-S ^b^	+	0.89 (0.01) [0]	0.08 (<0.0001) [0]
sR-38339-pE-cII-cy2001 in −10 *pE* (p765)	0-S ^b^	+	0.69 (<0.0001) [0]	0.01 (<0.0001) [0]
sR-38339-pE-cII-cy42 in −35 *pE* (p764)	0-S ^b^	+	1.00 (0.020) [0]	1.00 (<0.0001) [42]
sR-38339-pE-cII-cy3001 in −35 *pE* (p766)	H	−	0.72 (<0.0001) [0]	<0.001 (0.001) [8]
cII-oop-pO45WT (p763)	H	−	0.72 (0.10) [0]	0.07 (<0.0001) [0]
cII-oop-pO45-38,683-87MH2 in −10 *pO* (p759)	H	−	0.67 (0.10) [0]	<0.001 (0.002) [100]
cII-oop-pO45-38683LK in −10 *pO* (p762)	H	−	0.78 (0.20) [0]	<0.001 (<0.0001) [50]
cII-oop-pO94-38683-87MH (p681) ^c^	H	−	0.71 (<0.0001) [0]	<0.001 (<0.0001) [86]

^a^ Designations as in Table 2. ^b^ 0-S = Phage lysis spots/plaque areas were weak-turbid at 39 °C but well-formed clear plaques were observed at 37 °C. ^c^ Sequence change determined in putative −10 region of *pO* recovered from p27R*pO*^−^ [39] was attatG to cgcgcG and was identical to the synthetic change introduced into p759, see Figure S1, plasmids 2. ^d^ Individual plaques were clear.

## Supplementary Materials

Supplementary materials can be found at http://www.mdpi.com/1999-4915/10/3/115/s1.
