# Supplementary material for: The Bacteriophage Lambda CII Phenotypes for Complementation, Cellular Toxicity and Replication Inhibition Are Suppressed in cII-oop Constructs Expressing the Small RNA OOP
Source: Viruses. 2018 Mar 7;10(3):115. doi: 10.3390/v10030115 (PMC5869508; doi:10.3390/v10030115)
Supplement: Supplementary file 1 [file viruses-10-00115-s001.pdf]

Title: The Bacteriophage Lambda CII Phenotypes for Complementation, Cellular Toxicity, and Replication Inhibition Are Suppressed in *cII-oop* Constructs Expressing the Small RNA OOP.

Author List and Affiliations: Karthic Rajamanickam and Sidney Hayes, Department of Microbiology and Immunology, College of Medicine, University of Saskatchewan, Saskatoon, SK S7N 5E5 Canada

Corresponding author: sidney.hayes@usask.ca

Back Matter – Supplementary Materials

Figure S1. Sequence variations (1-3) of plasmid [cII_{1-97}] = [cII].

Figure S2. Deletions in *cII* and host mutations influencing CII complementation *in trans*.

Figure S3. Influence of *pO* variations on CII complementation *in trans*.

Table S1. Gene expression units cloned within plasmid vector pcIpR-[]-timm.

Table S2. Oligonucleotides used for plasmid construction and sequencing.

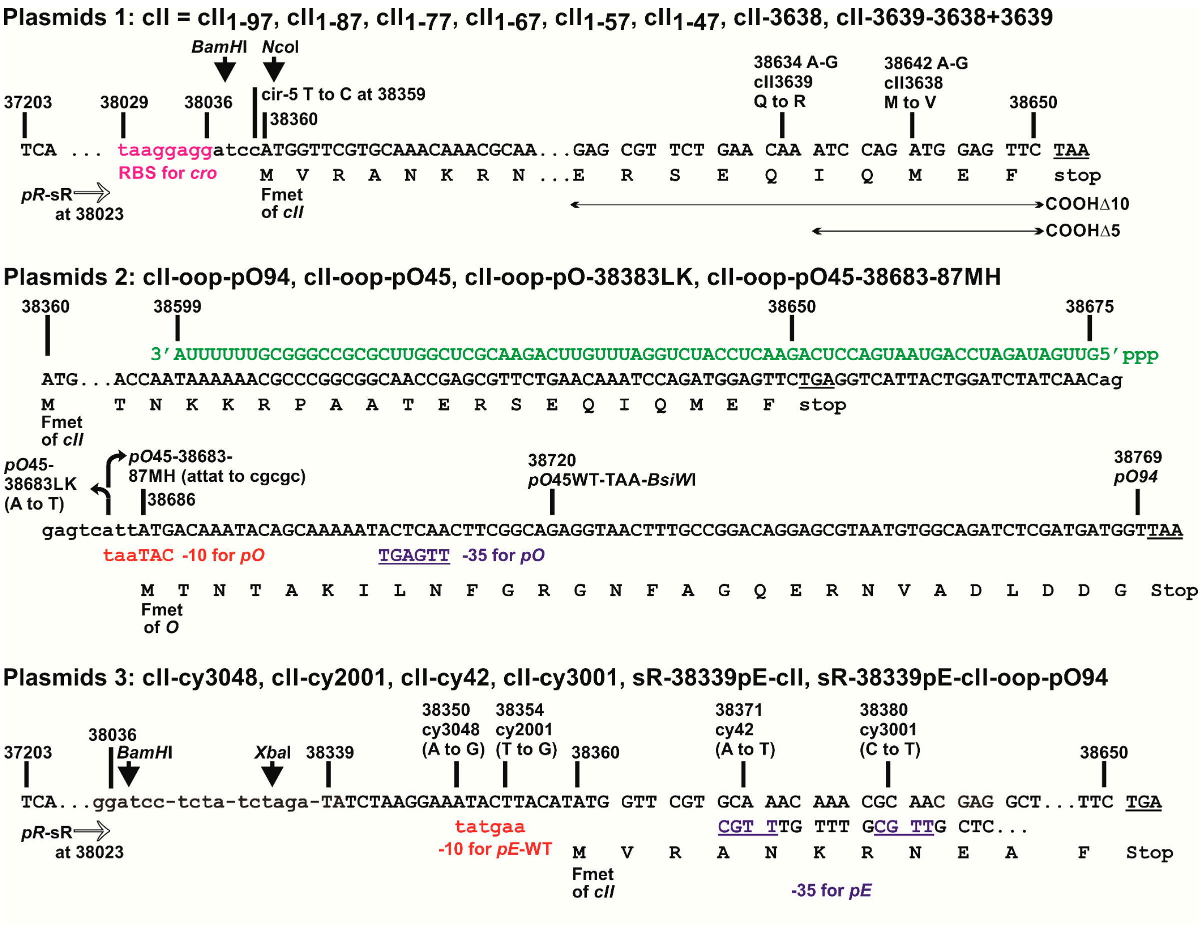

Figure S1. Sequence variations (1-3) of pcIpR-cII-timm enabling *cII* expression arrangements. The point mutations incorporated within *cII* or promoter *pO* were described in [1-4]. The sequences in *magenta* are RBS. For the plasmids in sequences 1 & 2 the RBS is identical to 8 bases of the RBS consensus sequence for gene *cro*. Sequences in *red* are "-10" promoter regions (for *pO*, in plasmid sequence 2; for *pE*, in plasmid sequence 3). The sequences in *blue* (underlined) are for "-35" region of *pO* promoter (sequence 2) and the suggested -35 region for the *pE* promoter (in sequence 3) within the N-terminal sequence of *cII*. The sequence in *green* (shown sequence 2) represents the *oop* sequence encoding OOP RNA. The Plasmids 1 include: cII, cII_{1-92} (deleting five amino acids from the carboxy-terminal end of *cII*), cII_{1-}87, cII_{1-77}, cII_{1-67}, cII_{1-57}, cII_{1-47}, cII-3638, cII-3639, cII-3638+3639. The sequence for Plasmids 2 include the sequence 1 for WT *cII* plus the *oop* sequence that overlaps the carboxyl end of *cII*, plus the *pO* sequence that overlaps with the sequence of N-terminal end of gene *O*: cII-oop-pO94, cII-oop-pO45, cII-oop-pO45-38383LK, cII-oop-pO-38683-87MH. The sequence for Plasmids 3 include the WT *pE* promoter from sR-38339-pE-cII, sR-38339pE-cII-oop-pO94 and *pE* mutations cII-cy3048, cII-cy2001, cII-cy42, and cII-cy3001.

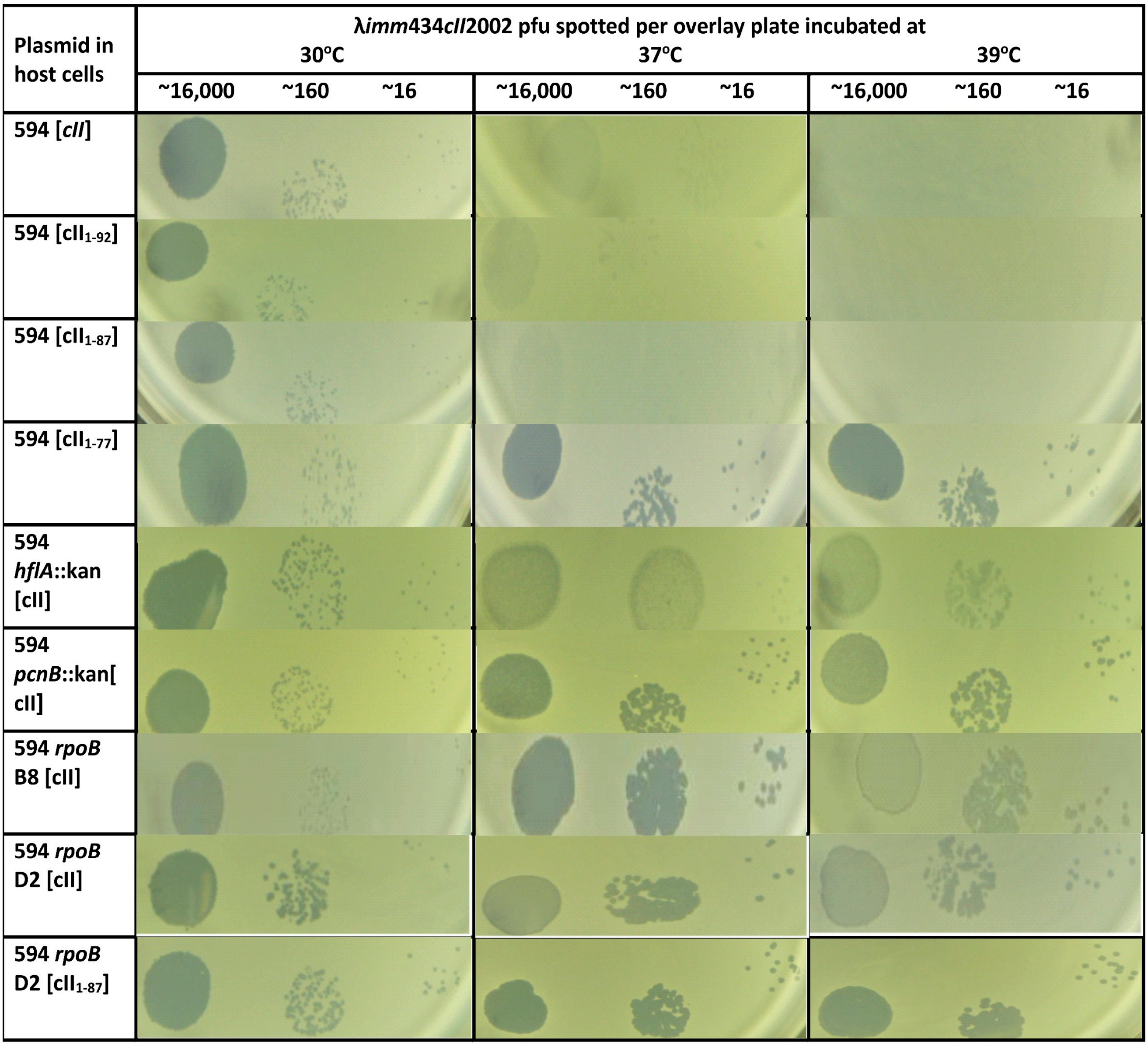

Figure S2. Deletions in *cII,* or host mutations influencing CII complementation *in trans*. Complementation of the *cII*-defect on the phage by the *cII* allele expressed from the plasmid was assessed by the ability of the spotted phage to form clear, turbid, or no pfu at 37 and 39°C. High expression of the CI_{434} repressor made from the phage will fully repress its *o*L (not assayed) and *o*R transcription, preventing phage growth (pfu formation). An intermediate level of *cII* expression from the plasmid at 37°C can permit the otherwise clear plaque forming λ*imm*434 *cII*68 and λ*imm*434*cII*2002 phages (seen at 30°C) to form turbid plaques at 37°C. In contrast, the expression of the *cII* allele on the plasmid is blocked when the Ts CI857 repressor is active in cells grown at 30°C.

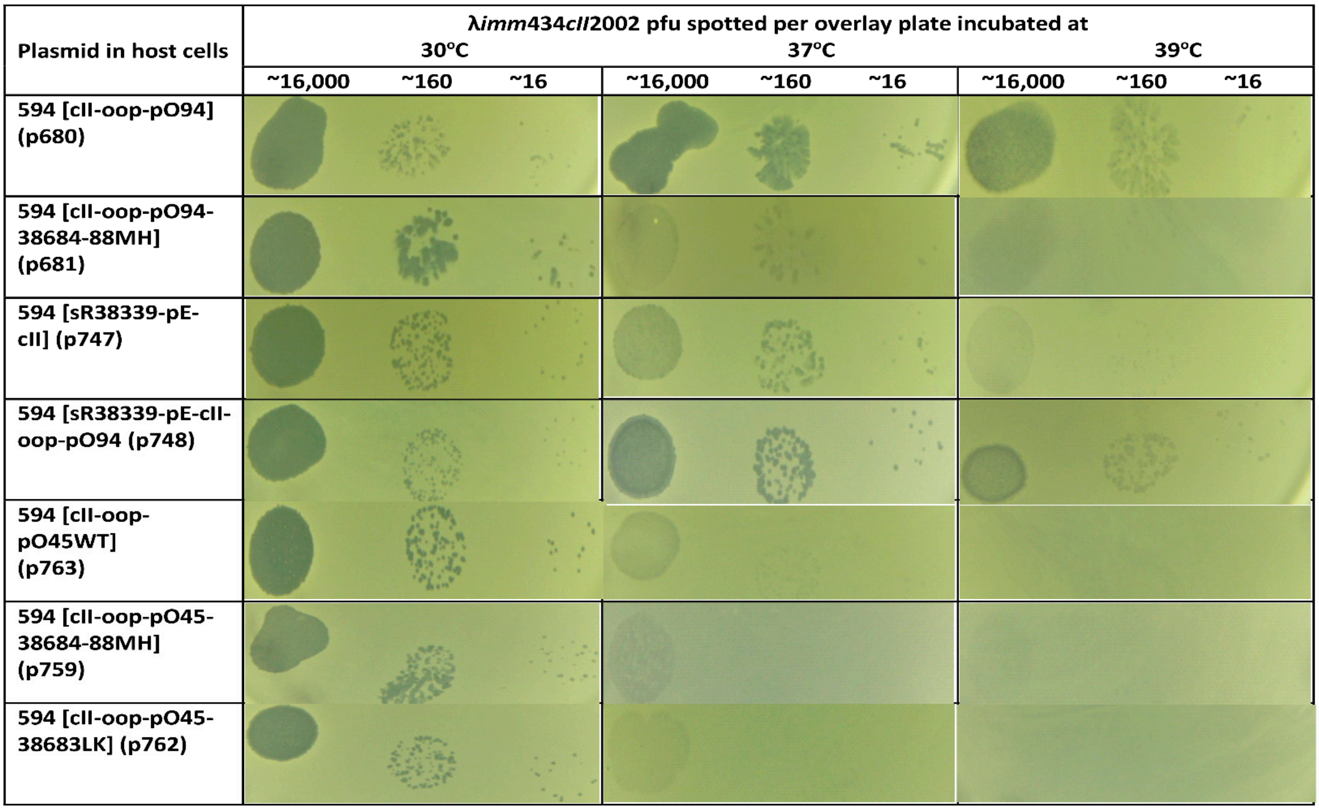

Figure S3. Influence of *pO* variations on CII complementation *in trans*.

Supplemental tables

Table S1. Gene expression units cloned within plasmid vector pcIpR-[]-timm.

Plasmid	Strain	Construction primer/ method employed
cII	p679	L-Bam-cII and R-ClaBsi-cII copy from p27R
cII-oop-pO94	p680	L-Bam-cII and R-Bsi-O-Po copy from p27R
cII-oop-pO94-38683-87MH	p681	L-Bam-cII and R-Bsi-O-Po copy from p27RpO$^-$
cII$_{1-92}$	p696	L-Bam-cII and R-Cla-Bsi-CIIΔ15
cII$_{1-87}$	p732	L-Bam-cII and R-Cla-Bsi-CIIΔ30
cII$_{1-77}$	p715	L-Bam-cII and R-Cla-Bsi-CIIΔ60
cII$_{1-67}$	p721	L-Bam-cII and R-Cla-Bsi-CIIΔ90
cII$_{1-57}$	p723	L-Bam-cII and R-Cla-Bsi-CIIΔ120
cII$_{1-47}$	p724	L-Bam-cII and R-Cla-Bsi-CIIΔ150
cII-oop-pO45WT	p763	L-Bam-cII and R-Bsi45poWT
cII-oop-pO45-38383LK	p762	L-Bam-cII and R-Bsi45po38383LK
cII-oop-pO45-38683-87MH	p759	L-Bam-cII and R-Bsi45po38684-88MH
cII-3638	p756	L-Bam-cII and R-Bsi-cII-3638
cII-3639	p757	L-Bam-cII and R-Bsi-cII-3639
cII-3638+3639	p758	L-Bam-cII and R-Bsi-3638+3639
sR-38339-cII-cy3048	p756	LXbaI-38339-cy3048 and R-ClaBsi-cII
sR-38339-cII-cy2001	p765	LXbaI-38339-cy2001 and R-ClaBsi-cII
sR-38339-cII-cy42	p764	LXbaI-38339-cy42 and R-ClaBsi-cII
sR-38339-cII-cy3001	p766	LXbaI-38339-cy3001 and R-ClaBsi-cII
sR-38339-pE-CII	p747	LBamXba38339 and R-nopo-Bsi-Cla (with WT cII stop)
sR-38339-pE-cII-oop-pO94	p748	LBamXba38339 and R-Bsi-O-po (with WT cII stop)

Table S2. Oligonucleotides used for plasmid construction and sequencing.

Primer Name	5' to 3' Sequence
L37904+18	gctgctcttgtgttaatgg
R-153-19	gaagacagtcataagtgcgg
L-Bam-CII	atatggatccatggttcgtgcaaacaaacgcaacgaggctctacg
R-ClaBsi-CII	atatatcgatcgtacgttagaactccatctggatttgttcagaacgctcgg
R-Bsi-45poWT	atat cgtacgttactgccgaagttgagtatttttgctgtatttgtcataatgactcctgt
R-Bsi-45po38383LK	atatcgtacgttactgccgaagttgagtatttttgctgtatttgtcataaAgactcctgt
R-Bsi-45po38684-88MH	atatcgtacgttactgccgaagttgagtatttttgctgtatttgtcGCGCGgactcctgt
R-Cla-Bsi-CII	atatatcgatcgtacgttagaactccatctggatttgttcagaacgctcgg
R-Cla-Bsi-CIIΔ15	atatatcgatcgtacgttattgttcagaacgctcggttgccgccgggcgtttttt
R-Cla-Bsi-CIIΔ30	atatatcgatcgtacgttaggttgccgccgggcgtttttttattggtgagaatcgc
R-Cla-Bsi-CIIΔ60	atatatcgatcgtacgttaaatcgcagcaacttgtcgcgccaatcgagccatgtc
R-Cla-Bsi-CIIΔ90	atatatcgatcgtacgttacatgtc gtcgtcaacgaccccccattcaagaacagc
R-Cla-Bsi-CIIΔ120	atatatcgatcgtacgttaaacagcaagcagcattgagaactttggaatccagtc
R-Cla-Bsi-CIIΔ150	atatatcgatcgtacgttaccagtccctcttccacctgctgatctgcgacttatc

**L-BamXba38339new**	**atatggatcctctatctagatatctaaggaaatacttacatatggttcgtgc**
**R-noPo-Bsi-ClaI**	**atatatcgatcgtacgtcagaactccatctggatttgttcagaacgctcggt**
**R-Bsi-O-Po**	**atatcgtacgttaaccatcatcgagatctgccacattacgctcctgtccggc**
**R-Bsi-CII-3638**	**atatcgtacgttagaactccaCctggatttgttcagaacgctcgg**
**R-Bsi-CII-3639**	**atatcgtacgttagaactccat ctggattCgttcagaacgctcgg**
**R-Bsi-3638+3639**	**atatcgtacgttagaactccaCctggattCgttcagaacgctcgg**
**LXbaI-38339-cy3048**	**atattctagatatcttaggaaGtacttacatatggttcg**
**LXbaI-38339-cy2001**	**atattctagatatcttaggaaatacGtacatatggttcgtgc**
**LXbaI-38339-cy42**	**atattctagatatcttaggaaatacttacatatggttcgtgcTaacaaacgcaacgaggc**
**LXbaI-38339-cy3001**	**atattctagatatcttaggaaatacttacatatggttcgtgcaaacaaacgTaacgaggctctacgaatc**
**L-Bam-OOP#1**	**atatggatcctggctcgattggcgcgacaagt**
**L-Bam-OOP#2**	**atatggatccgttgacgacgacatggctcgat**
**R-Cal-O**	**atatatcgattatagatccaccccgtaaatccagtc**
**R-ClaI-36P**	**atatatcgattacctgctgtacctgcggcttttcgtcg**
**R-ClaI-63P**	**atatatcgattacttcgttctggtcacggttagcc**

References

1. Daniels, D. L., Schroeder, J.L., Szybalski, W., Sanger, F., and Blattner, F.R, Appendix I. A molecular map of coliphage lambda. In *Lambda II*, R.W. Hendrix, J. W. R., F.S. Stahl, R.A. Weisberg, Ed. Cold Spring Harbor Laboratory: Cold Spring Harbor, New York, 1983; pp 469-517.
2. Hayes, S.; Horbay, M. A.; Hayes, C., A CI-Independent Form of Replicative Inhibition: Turn Off of Early Replication of Bacteriophage Lambda. *PLoS One* **2012,** 7, (5), e36498.
3. Krinke, L.; Mahoney, M.; Wulff, D. L., The role of the OOP antisense RNA in coliphage lambda development. *Mol Microbiol* **1991,** 5, (5), 1265-72.
4. Wulff, D. L., Rosenberg, M.R., Establishment of repressor synthesis. In *Lambda II*, Hendrix, R. W., Roberts, J.W., Stahl, F.W., and Weisberg, R.A., Ed. Cold Spring Harbor Laboratory Press: Cold Spring Harbor, NY, 1983; pp 519-684.
